# Lachesis Trigonocephalus—Proving by Induction of the 7m. Fincke

**Published:** 1885-11

**Authors:** 

**Affiliations:** Alvensleben, Germany


### LACHESIS TRIGONOCEPHALUS—PROVING BY INDUCTION OF THE 7M. FINCKE.

DR. BUCHMANN, ALVENSLEBEN, GERMANY.

Ernest Wagner, seventeen years, student, had, when a child, spasmus glottidis. Two years ago a bronchial catarrh was accompanied by asthmatic attacks in the night, which returned several times, and which, for the last time before Easter of this year, had caused three successive sleepless nights.

1885, July 14th, 8.10 A.M.—Prover took the vial with Lachesis 7M. (F.) for fifteen minutes in his right hand, sitting on the sofa.

8.15 A. M.—Yawning, frequently repeated; accumulation of water in the mouth, watering eyes.

8.25 A. M.—Tickling in the nostrils and watery secretion in the nasal cavity. Aching between the shoulder blades, lasting five minutes; sensation of firmly adhering mucus in the pharynx.

Rumbling below the navel. The yawning continues. Great tiredness and indisposition to mental labor. The eyes get watery again.

10 A. M.—The tiredness has ceased. The voice has a crowing character which strikes him very much. In the afternoon, increased thirst.

Toward 5 P. M., very much depressed. The crowing voice gets worse toward evening. Immediately after supper the tiredness increased.

July 15th.—In spite of great tiredness, difficulty to fall asleep. Before midnight, waking up from sleep frightened, after which he falls soon asleep again three times. On rising in the morning he notices a great spot in the cheek—a thing which never happened before and frightens him considerably. A seminal emission occurred during sleep without his knowledge, without erection, and unaccompanied by dream. After getting up, some transient dyspnœa.

July 16th.—After going to bed at 9 P. M., being very tired, itching on the left instep near the toes, then upon the left shoulder near the neck, then upon the left hand near the fingers, then upon the left side of his face, especially at the left side of the nose, then at the left ear-lap as far as the lower part of the cartilage of the concha, then outside of the leg near the left tibia. Now and then the itching returned at these parts, causing him to scratch till 10 P. M., when he fell asleep. He has never observed a similar itching before in his life. During the day, voice somewhat rough.

July 18th, 6.10 A. M.—The prover took the same vial in the left hand for half an hour. After about ten minutes, yawning several times, watering eyes, sensation of mucus adhering to the pharynx, tiredness.

After removing the vial no more symptoms were observed.

---

It will be seen that this proving furnishes interesting symptoms which correspond with Hering's proving of the thirtieth cent. Interesting, also, is the comparative immunity to Lachesis on a second application, after cessation of all the symptoms, and the following short duration of action (Wirkungsdauer).

---

THE SOUTHERN JOURNAL OF HOMŒOPATHY: *The Southern Hom. Pellet* comes out under this new name, in a new dress and with brighter pages. Success to it, and may it do much good in the Southern land.

